# Clavine Alkaloids Gene Clusters of *Penicillium* and Related Fungi: Evolutionary Combination of Prenyltransferases, Monooxygenases and Dioxygenases

**DOI:** 10.3390/genes8120342

**Published:** 2017-11-24

**Authors:** Juan F. Martín, Rubén Álvarez-Álvarez, Paloma Liras

**Affiliations:** Department of Molecular Biology, Section of Microbiology, University of León, 24071 León, Spain; ralva@unileon.es (R.Á.-Á.); paloma.liras@unileon.es (P.L.)

**Keywords:** clavine alkaloids, ergot alkaloids, fumigaclavine, isofumigaclavine, agroclavine, festuclavine, cycloclavine synthase, phytanoyl-CoA hydroxylase, *Penicillium roqueforti*, *Neosartorya fumigata*

## Abstract

The clavine alkaloids produced by the fungi of the Aspergillaceae and Arthrodermatacea families differ from the ergot alkaloids produced by *Claviceps* and *Neotyphodium*. The clavine alkaloids lack the extensive peptide chain modifications that occur in lysergic acid derived ergot alkaloids. Both clavine and ergot alkaloids arise from the condensation of tryptophan and dimethylallylpyrophosphate by the action of the dimethylallyltryptophan synthase. The first five steps of the biosynthetic pathway that convert tryptophan and dimethylallyl-pyrophosphate (DMA-PP) in chanoclavine-1-aldehyde are common to both clavine and ergot alkaloids. The biosynthesis of ergot alkaloids has been extensively studied and is not considered in this article. We focus this review on recent advances in the gene clusters for clavine alkaloids in the species of *Penicillium*, *Aspergillus* (*Neosartorya*), *Arthroderma* and *Trychophyton* and the enzymes encoded by them. The final products of the clavine alkaloids pathways derive from the tetracyclic ergoline ring, which is modified by late enzymes, including a reverse type prenyltransferase, P450 monooxygenases and acetyltransferases. In *Aspergillus japonicus*, a α-ketoglutarate and Fe^2+^-dependent dioxygenase is involved in the cyclization of a festuclavine-like unknown type intermediate into cycloclavine. Related dioxygenases occur in the biosynthetic gene clusters of ergot alkaloids in *Claviceps purpurea* and also in the clavine clusters in *Penicillium* species. The final products of the clavine alkaloid pathway in these fungi differ from each other depending on the late biosynthetic enzymes involved. An important difference between clavine and ergot alkaloid pathways is that clavine producers lack the enzyme CloA, a P450 monooxygenase, involved in one of the steps of the conversion of chanoclavine-1-aldehyde into lysergic acid. Bioinformatic analysis of the sequenced genomes of the Aspergillaceae and Arthrodermataceae fungi showed the presence of clavine gene clusters in *Arthroderma* species, *Penicillium roqueforti*, *Penicillium commune*, *Penicillium camemberti*, *Penicillium expansum*, *Penicillium steckii* and *Penicillium griseofulvum*. Analysis of the gene clusters in several clavine alkaloid producers indicates that there are gene gains, gene losses and gene rearrangements. These findings may be explained by a divergent evolution of the gene clusters of ergot and clavine alkaloids from a common ancestral progenitor six genes cluster although horizontal gene transfer of some specific genes may have occurred more recently.

## 1. Clavine and Ergot Alkaloids: An Overview

**Figure 1:**
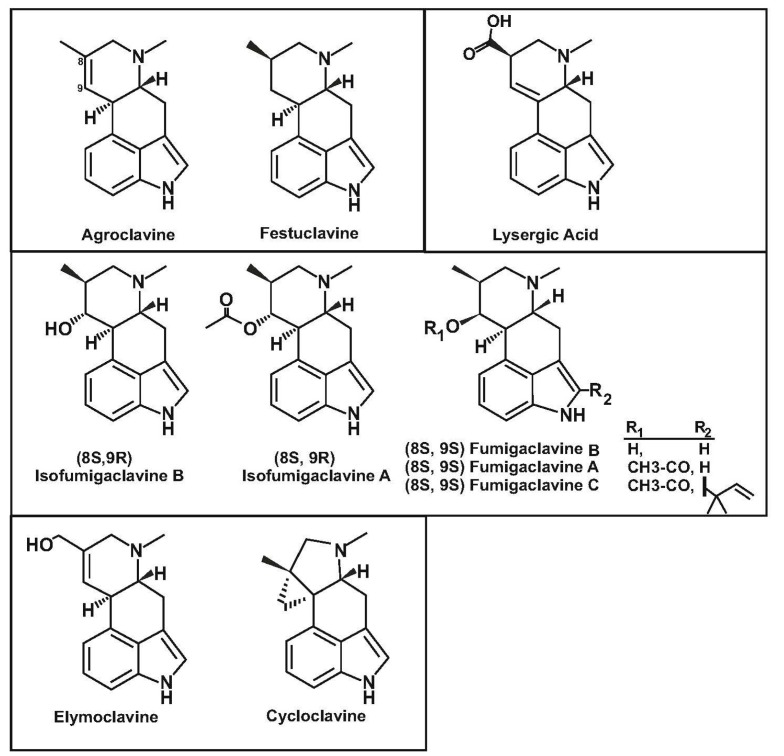
The chemical structures of agroclavine, festuclavine and lysergic acid (**upper panel**). (8S, 9R) isofumigaclavines A and B and (8S, 9S) fumigaclavines A, B and C (**middle panel**). Elymoclavine and cycloclavine (**lower panel**).

There are two clearly distinct types of ergot alkaloids. The simple clavine type alkaloid produced by fungi of the Aspergillaceae and Arthrodermataceae families (Figure 1) and the lysergic acid-derived ergot alkaloids produced by parasitic or endophytic Clavicipitaceae fungi. The clavine alkaloids—hereafter named simply clavines—contain a tetracyclic ergoline nucleus with minor modifications (hydroxylations, acetylations, prenylations, methylations, cyclopropane ring formation) [1,2]. On the other hand, the classical lysergic (or paspalic) acid-derived ergot alkaloids contain large modifications of the ergoline scaffold and are subdivided in two groups: the ergoamides and the ergopeptines. These two groups differ in the fact that the ergopeptines contains a variety of peptide substituents synthesized by non-ribosomal peptide synthetases (NRPS), encoded by genes which are linked to the ergoline nucleus biosynthetic genes, whereas d-lysergic acid is linked to a 2-aminoalcohol by an amide linkage in the ergoamides. 

**Figure 2:**
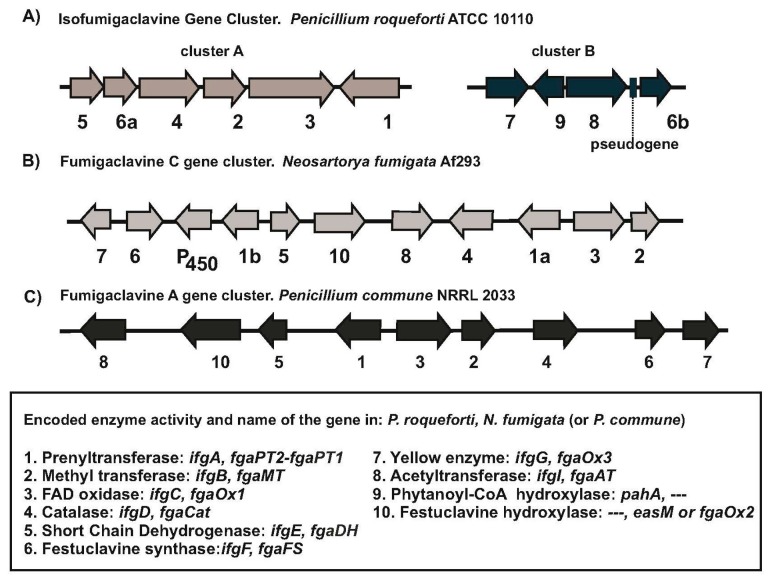
Organization of the clavine gene clusters. (**A**) Clusters A and B of Isofumigaclavine in *P. roqueforti*; (**B**) Fumigaclavine C gene cluster of *N. fumigata*. (**C**) Fumigaclavine A gene cluster of *P. commune*. In the lower panel the numbers correspond to the enzymatic activities encoded by the genes, followed by the gene names in *P. roqueforti* and *N. fumigata*/*P. commune*. The symbol “---” indicates that the corresponding gene is not present in the published cluster.

Clavine and lysergic acid-containing alkaloids derive from the condensation of tryptophan and dimethylallylpyrophosphate (DMA-PP), catalyzed by the first enzyme of the pathway, named dimethylallyltryptophan synthase (DMATS). This results in the formation of tricyclic intermediates, the most relevant being chanoclavine I and chanoclavine I aldehyde, which is the branch point intermediate for the formation of different types of alkaloids [3,4]. Chanoclavine I aldehyde, is later cyclized to form the tetracyclic ergoline nucleus. The ergoline structure constitutes the nucleus of festuclavine, agroclavine, elymoclavine, pyroclavine and their derivatives (Figure 1). In agroclavine and elymoclavine, there is a double bond between carbons C-8 and C-9, whereas in festuclavine this double bond is not present. Pyroclavine, an C-8 stereoisomer (carbons 8R/9S) of festuclavine (carbon 8S/9S), is also present in cultures of some of the clavine alkaloids producers. The late steps of the biosynthesis of fumigaclavine alkaloids have been elucidated in *Neosartorya fumigata*. These steps involve the oxidation of festuclavine to 9-hydroxyfestuclavine (named fumigaclavine B) and later the acetylation of fumigaclavine B to fumigaclavine A (Figure 2) [5]. Finally, in *N. fumigata*, there is a step that involves the prenylation of fumigaclavine A to form fumigaclavine C. The intermediates fumigaclavine A and B have interesting potential as antileukemic agents [2]. Moreover, festuclavine is an interesting intermediate for the synthesis of dihydrolysergic acid derivatives, which have important medical applications.

In addition to the studies with *N. fumigata*, recently clavine gene clusters have been found in *Penicillium commune* [6], in *A. japonicus*, that forms cycloclavine [7,8], in several Arthrodermataceae family members [9] and recently in *Penicillium roqueforti* [10]. In this article, we focus our attention on a comparative study of the clavine gene clusters and their encoded enzymes in *Penicillium*, *Aspergillus* and related Aspergillaceae fungi in comparison with the better known lysergic acid-derived alkaloids produced by Clavicipitaceae fungi. The biosynthesis of ergot alkaloids in *Claviceps* and *Epichlöe* species has been studied in detail because of their importance in animal and human toxicosis and the use of ergot alkaloid derivatives in medicine [11,12,13,14] and, therefore, is not considered here.

## 2. The Clavine Gene Cluster in *Penicillium roqueforti*: Comparison with *Neosartorya fumigata*

*P. roqueforti* is known to produce isofumigaclavine A and isofumigaclavine B, isomers 8S/9R of fumigaclavine A and B, respectively [15,16]. In addition, many natural strains of *P. roqueforti* accumulate the intermediates festuclavine and agroclavine [17]. *P. roqueforti* is used industrially as inoculum for ripening cheeses and it is known to produce a variety of secondary metabolites [18,19]. Recently, the roquefortine gene cluster of *P. roqueforti* has been characterized [20]. During the study of a novel dimethylallyltryptophan synthase gene in *P. roqueforti*, different from the roquefortine prenyltransferase, Fernández Bodega and coworkers [10] found that silencing of this second DMATS gene, blocked the biosynthesis of isofumigaclavine A but not that of roquefortine C. These studies indicated that this second DMATS gene of *P. roqueforti*, named *ifgA*, is involved in the biosynthesis of isofumigaclavine A. In addition, silencing of the *ifgA* gene blocked or reduced the biosynthesis of two other compounds [10] that are likely prenylated intermediates or oxidized products derived of agroclavine [1,2] but does not affect the production of mycophenolic acid or andrastin A. Interestingly, the formation of roquefortine C was increased 30% in the *ifgA* silenced mutant, suggesting that blocking the clavine pathway diverts the precursors tryptophan and DMA-PP to form roquefortine C. The *ifgA* gene, encodes a 462 amino acids DMATS enzyme of the direct (normal) type [21]. Bioinformatic analysis showed that this protein is highly similar to other DMAT synthases in *Penicillium camemberti*, *Penicillium expansum*, *Penicillium steckii*, *Penicillium griseofulvum* and *P. commune* (see below). *N. fumigata* contains two prenyltransferases [14,22,23,24] in the fumigaclavine gene cluster, FgaPT2 and FgaPT1, of which FgaPT2 catalyzes the first step of the fumigaclavine biosynthetic pathway. The *P. roqueforti ifgA* encoded protein is similar to the FgaPT2 of *N. fumigata* (63% identity) and this suggests that IfgA is the first enzyme of the clavine biosynthetic pathway in *P. roqueforti*. The FgaPT1 enzyme is a reverse prenyltransferase that introduces a dimethylallyl group at the C-2 carbon of fumigaclavine A to form fumigaclavine C; this reverse prenyltransferase is absent in the genome of *P. roqueforti* and therefore this fungus is able to form isofumigaclavine A but does not convert this product to isofumigaclavine C [10]. On the other hand, the IfgA protein is more distantly related to DmaW, the *Claviceps purpurea* homologous protein (58% identity), what suggests interesting implications in the evolution of these genes (see below).

### 2.1. The Clavine Biosynthetic Genes of Penicillium roqueforti Are Located into Two Clusters

**Figure 3:**
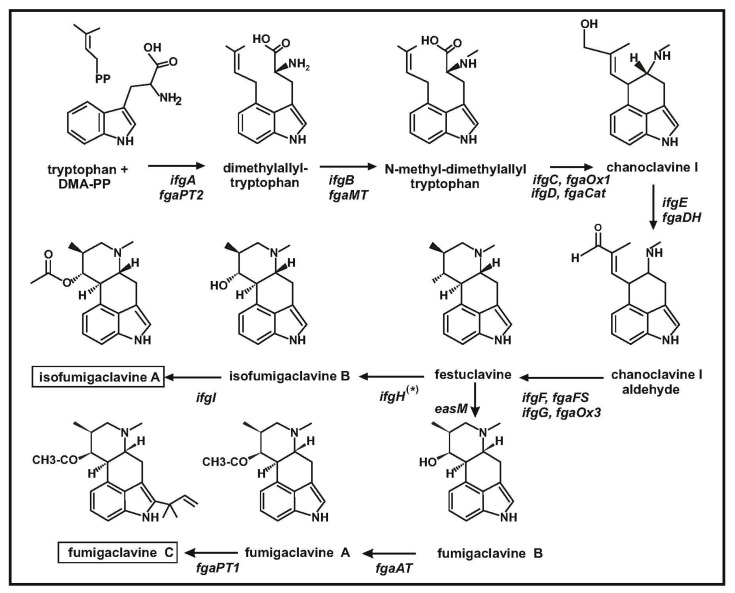
Biosynthetic pathway of isofumigaclavine A in *P. roqueforti* and fumigaclavine C in *N. fumigata*. The *ifgH* gene for the festuclavine hydroxylase, labelled with an asterisk, is not located in any of the two isofumigaclavine clusters of *P. roqueforti*. The names of the genes in *N. fumigata* are indicated below the names of the *P. roqueforti* genes.

Analysis of the genes adjacent to *ifgA* in the genome of *P. roqueforti* revealed that there are six linked gene, named *ifgA* to *ifgF1* (cluster A), which are involved in the conversion of tryptophan into festuclavine. The organization of these six genes is shown in Figure 2A. The functionality of *ifgA* has been proved by RNA silencing [10]. The role of the proteins encoded by the other genes has been proposed by comparison with the enzymes encoded by the orthologous genes of *N. fumigata*, characterized by gene disruption, gene complementation and in vitro heterologous expression [14,25] (Table 1). The second gene in the pathway is *ifgB* encoding the *N*-methyltransferase [26], which introduces a methyl group in the amino group of DMAT (Figure 3). The *ifgC* and *ifgD* genes encode a FAD-dependent oxidoreductase and a catalase-like enzyme, respectively, acting together in the conversion of *N*-methyl-DMAT to chanoclavin I [27]. The catalase-like enzyme, IfgD, is likely to be localized in peroxisomes since it contains a peroxisomal targeting sequence (PTS1) of *N. fumigata* catalase is not essential for enzyme activity when the gene is expressed heterologously in *Saccharomyces cerevisiae* [28]. The chanoclavine I is converted in chanoclavine I aldehyde by the short chain dehydrogenase encoded by the following gene, *ifgE* [29]. The enzymatic activity of the *P. roqueforti* short chain dehydrogenase has been confirmed in vitro using purified enzyme [30]. The sixth gene in the cluster, *ifgF1*, encodes a festuclavine synthase that is involved in the conversion of chanoclavine I aldehyde into festuclavine. 

Surprisingly, cluster A do not contain a gene homologous to that encoding the FMN-containing old yellow enzyme (FgaOx3 in *N. fumigata*) that is well known to be required for the formation of fumigaclavine in *N. fumigata*. The absence of a gene for the yellow enzyme was clarified when a homologous gene (67% identity to FgaOx3) was found in a second location (cluster B) in *P. roqueforti* genome. Cluster B contains four genes related to the late steps of isofumigaclavine biosynthesis (Figure 3, Table 1). In addition to the *ifgG* gene for the yellow enzyme, cluster B contains a second festuclavine synthase gene, *ifgF2* (77% identity to IfgF1) and a gene for an acetyltransferase (*ifgI*), putatively involved in the conversion of isofumigaclavine B to isofumigaclavine A. A fourth gene, named *pahA*, encodes a phytanoyl-CoA hydroxylase-like enzyme. Enzymes related to the phytanoyl-CoA hydroxylase have been found in alkaloid producing fungi such as the cycloclavine synthase, involved in the cyclization of chanoclavine I aldehyde to cycloclavine in *A. japonicus* and the hydroxylase that convert ergotamam to ergotamine in *C. purpurea* (see below). 

None of the 10 genes located in clusters A and B (9 genes and a duplicated *ifgF* gene), corresponds to an orthologue of the festuclavine hydroxylase, a cytochrome P450 that has been shown to catalyze in *N. fumigata* the conversion of festuclavine into fumigaclavine B by hydroxylation of C-9 [5]. Alternative genes for P450 cytochrome monooxygenases were found in *P. roqueforti* genome (CDM27384 and CDM36212, with 41% and 35% identity to *N. fumigata* EasM, also named FgaOx2) but the implication of these alternative P450 monooxygenases in the hydroxylation at C-9 has not been studied. Accumulation of prenylated intermediates and lack of fumigaclavine A formation has been reported in mutants of *N. fumigata* disrupted in the festuclavine hydroxylase [5]. Similarly, the lack of an authentic orthologous festuclavine hydroxylase in *P. roqueforti* may explain the observed accumulation of similar high molecular weight compounds [10] and possibly also of the earlier intermediates agroclavine and festuclavine [17].

### 2.2. Two Yellow Enzymes Genes Exist in Penicillium roqueforti and Neosartorya fumigata

Interestingly, a second yellow enzyme in *P. roqueforti*, named FgaOx3**_Pr3_** (51% identity to IfgG), has been reported recently [30]. This second yellow enzyme, has been located in scaffold 3, outside of the two clavine clusters previously identified [10]. The yellow enzyme encoded by this gene is able to convert the substrate, chanoclavine I aldehyde, in combination with FgaFS into festuclavine. This enzyme is not able to produce festuclavine in absence of FgaFS, as reported also for all studied yellow enzymes. In absence of the FgaFS the new yellow enzyme stimulated drastically the conversion of chanoclavine I into chanoclavine I aldehyde, apparently by removing a product feedback inhibition of the chanoclavine I dehydrogenase encoded by the *ifgE* gene. The new enzyme FgaOx3**_Pr3_** has lower similarity to the *N. fumigata* yellow enzyme FgaOx3 (52% identity) than the cluster encoded *P. roqueforti* IfgG (67% identity). This finding suggests that at least two different old yellow enzymes are acting in *P. roqueforti* and that both of them may contribute to isofumigaclavine biosynthesis although the mechanisms of these two enzymes may be slightly different. At this time, it is unclear whether the *ifgG* encoded protein has also a stimulatory effect on the chanoclavine I dehydrogenase. 

Worth noting, a second yellow enzyme of *N. fumigata*, that we name FgaOx4 (XP_749538) is highly similar to FgaOx3**_Pr3_** (71%) described in *P. roqueforti* [30]. The role of this second yellow enzyme, that is located outside of the fumigaclavine gene cluster is unclear, since previous studies of mutant disrupted in the cluster located *fga*Ox3 [3] showed that the disrupted mutant is unable to produce festuclavine and fumigaclavines A, B and C. This mutant accumulates chanoclavine I and chanoclavine I aldehyde. The involvement of FgaOx3 in the biosynthesis of fumigaclavine has been confirmed by in vitro studies using purified enzyme in combination of the partner enzyme FgaFS [31]; therefore, the role of FgaOx4 in the biosynthesis of fumigaclavine is doubtful. Several old yellow enzymes are known to be involved in the reduction of the double bond conjugate to aldehyde or ketone groups in different molecules and are used industrially for the reduction of different alkemes. The FgaOx3**_Pr3_** has been annotated as *N*-ethylmaleimide reductase [32]. Therefore, it is likely that its major role is different from that of clavine biosynthesis. 

### 2.3. Penicillium roqueforti Synthesize Both Festuclavine and Agroclavine: The Role of Tyrosine versus Phenylalanine in the Yellow Enzyme Active Center

In addition to festuclavine, *P. roqueforti* forms agroclavine, a molecule similar to festuclavine but carrying a C-8 to C-9 double bond, which is not formed in detectable amounts by *N. fumigata*. 

In *N. fumigata*, the C8–C9 double bond in chanoclavine aldehyde is reduced by the yellow enzyme FgaOx3 (EasA, reductase-type) and the FgaFS, allowing the aldehyde group free rotation and its interaction with the secondary amine to promote the ring closure of the fourth ring via a Schiff base formation [11,14,29].

However, most ergot alkaloid producing fungi in the Clavicipitaceae family, synthesize the 8, 9 unsaturated clavine agroclavine from chanoclavine aldehyde via the activity of an alternative version of EasA that acts as an isomerase rather than as a reductase [3].

**Figure 4:**
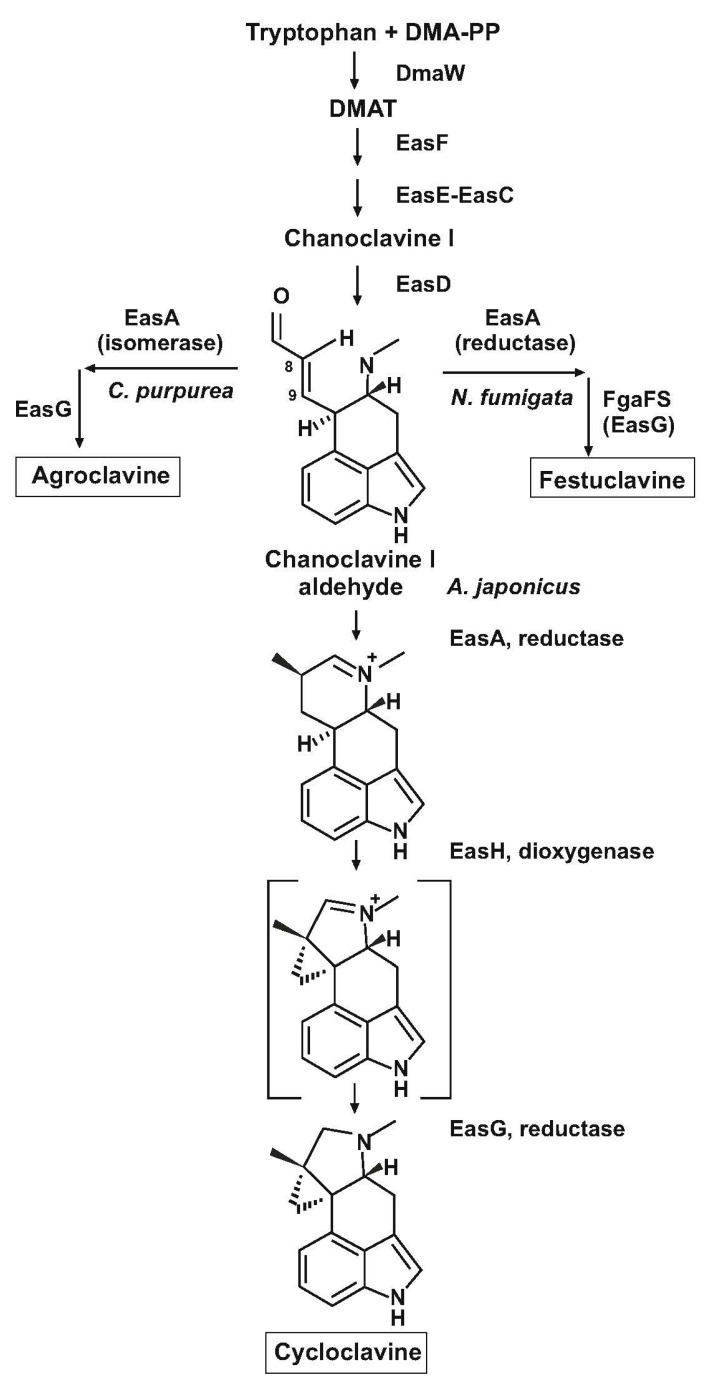
Biosynthetic pathway of cycloclavine in *A. japonicus*. The intermediate compound in brackets has been proposed by Jakubczyk et al. [7,8]. On each side the branches leading to agroclavine and festuclavine in *C. purpurea* and *N. fumigata*, respectively, are shown.

Furthermore, two enzymes agroclavine synthase (EasG) in *Claviceps* and festuclavine synthase (FgaFS) in *N. fumigata*, that perform similar enzymatic reactions in combination with the yellow enzyme, resulting in the formation of agroclavine and festuclavine, respectively, have only a 46% identity, what agrees with the different products formed (Figure 4). Therefore, an interesting question is how *P. roqueforti* is able to form both agroclavine and festuclavine.

Another important finding in the *P. roqueforti* clavine cluster is the presence in the yellow enzyme of a tyrosine in the active center. The yellow enzyme in *N. fumigata*, as in the *P. roqueforti* enzyme, contains a tyrosine at position 181 (Y-181), whereas the yellow enzyme of *C. purpurea* shows a phenylalanine at this position [31]. The role of tyrosine/phenylalanine in the enzymatic activity of the yellow enzyme has been confirmed by in vivo gene disruption and in vitro enzymatic studies [3,29]. This tyrosine, apparently, acts as a proton donor during cyclization of the chanoclavine I aldehyde to form the tetracycle ergoline nucleus resulting in the formation of festuclavine [31]. The presence of Y-181 indicates that the yellow enzyme of *P. roqueforti* is of the reductase type, what is consistent with the formation of festuclavine and isofumigaclavine but it does not explain the reported formation of agroclavine in several different strains of *P. roqueforti*. It is likely that agroclavine is formed by non-enzymatic isomerization of the double bond between C-8 and C-9, in presence of reduced glutathione [33]. The second yellow enzyme FgaOx3**_Pr3_** of *P. roqueforti* also contains a tyrosine in the corresponding position [30]. We have compared the yellow enzymes of several *Penicillium* species and all of have a tyrosine in the equivalent position of the *N. fumigata* yellow enzyme active center.

### 2.4. A Fe^2+^ and α-Ketoglutarate-Dependent Dioxygenase Is Encoded in Cluster B of Penicillium roqueforti

In the last few years an interesting development is the finding that genes encoding phytanoyl-CoA hydroxylase-like enzymes are present in several clusters of clavine and ergot alkaloid in different fungi. These enzymes belong to the Fe^2+^ and α-ketoglutarate-dependent dioxygenases family but are not stimulated by ascorbic acid in contrast to some other O_2_-splitting oxygenases such as the isopenicillin N synthase [34,35]. These dioxygenases use molecular oxygen but introduce only one oxygen atom in their substrates; the other oxygen atom is accepted by α-ketoglutarate that is converted in CO_2_ and succinate. The phytanoyl hydroxylases form a group of dioxygenases divided in several families [36,37] with a wide spectrum of substrates. The model enzyme of this family is the human phytanoyl-CoA hydroxylase, Pahx, that has been widely studied and crystallized [38,39]. In *A. japonicus*, the cycloclavine synthase, EasH, belongs to the phytanoyl-CoA hydroxylase family. This enzyme contains the conserved HRE sequence characteristic of hydroxylases. Furthermore, an EasH protein similar to that of *A. japonicus* has been found to be involved in the hydroxylation of the late-stage ergot intermediate dihydroergotamam to dihydroergotamine in *C. purpurea* [37]. A detailed analysis of *C. purpurea* EasH showed that this enzyme introduces a hydroxyl group at the α-carbon (C-2) of alanine or valine, the first (proximal to lysergic acid) amino acids of the tripeptide chain of the ergopeptams intermediate which is later converted in ergopeptines. 

This hydroxylation reaction takes place in conjunction with the non-ribosomal peptide synthethases LPS1/LPS2 of *Claviceps* and results in the formation of the oxygen containing oxazolidine ring in the tripeptide chain of ergotamines.

The role of the phytanoyl-CoA hydroxylase-related gene in *P. roqueforti* is obscure because no specific reaction of the isofumigaclavine biosynthetic pathway has been shown to be catalyzed by this enzyme but it has important evolutionary implications that will be discussed at the end of this article.

## 3. A Fumigaclavine Gene Cluster in *Penicillium commune*

Taking into account the phylogenetic closeness between all *Penicillium* species it is likely that similar clavine alkaloid clusters occur in other *Penicillium* species, although it is well known that *Penicillium* species differ in the secondary metabolites that they produce. One of them is *Penicillium commune* NRRL2033 that is reported to synthesize fumigaclavine A [40]. A nine genes cluster for the biosynthesis of fumigaclavine was located by hybridization of a cosmid library with the DMAT synthase gene. This gene was cloned by PCR amplification using primers based on the *N. fumigata fgaPT2* gene [6,14]. Comparison of the available sequences indicates that the genes in *P. commune* clavine gene cluster are similar to those of *P. roqueforti* except that they are all linked in a single cluster [41]. Indeed, *P. commune* clavine gene cluster contains: (i) the five genes of the common core of all ergot alkaloid pathways that converts tryptophan into chanoclavine I aldehyde; (ii) two genes for the conversion of chanoclavine I aldehyde to festuclavine and; (iii) genes for the hydroxylation of festuclavine at C-9 and for the acetylation of fumigaclavine B to fumigaclavine A. Two of these genes, the yellow enzyme gene and the festuclavine synthase gene, were characterized in some detail [14,41]. Comparison of encoded proteins showed that the *P. commune* yellow enzyme and festuclavine synthase show 64% and 76% identity, to the homologous enzymes of *P. roqueforti*, respectively. The *P. commune* yellow enzyme contains a tyrosine at the same position of *P. roqueforti* and *N. fumigata* orthologous proteins. The *P. commune* clavine cluster lacks the gene *fgaPT1* converting fumigaclavine A into fumigaclavine C and therefore *P. commune* is unable to synthesize fumigaclavine C.

## 4. A Cycloclavine Gene Cluster in *Aspergillus japonicus* and *Byssochlamys spectabilis*

A new clavine, cycloclavine, different from the classical fumigaclavine was discovered in seed of *Ipomea hildebrandtii* [42]. The chemical structure of cycloclavine was confirmed in culture extracts of *A. japonicus* by ^1^H-NMR and ^13^C-NMR studies [7,43]. It shows differences with the structure of the ergoline scaffold in festuclavine or agroclavine, namely the carbons C-8, C-9 and C-10 in the structure of cycloclavine form a cyclopropane ring (Figure 1) in contrast to the ergoline nucleus, in which there is no cyclopropane ring. An internal cyclization step is involved in the formation of the cyclopropane ring of cycloclavine. In addition to cycloclavine *A. japonicus* produces festuclavine. A study of the *A. japonicus* genome allowed to clone a region of 16.8 kb that contains eight genes involved in the biosynthesis of cycloclavine [7] (Table 1).

Seven of these eight genes (*easA* to *easG*) correspond to the equivalent genes in the fumigaclavine gene cluster of *N. fumigata* and *P. roqueforti* and are also similar to the homologous genes in *C. purpurea*. These seven genes are involved in the conversion of tryptophan and DMA-PP, first to chanoclavine I aldehyde and then to an ergoline containing intermediate similar to festuclavine. The eighth gene, named *easH*, encodes a cycloclavine synthase that results in the formation of cycloclavine. The involvement of *easH* in clavine or ergot alkaloid biosynthesis had not been described before. 

The implication of the eight genes in the biosynthesis of cycloclavine was proved by expressing combinations of different sets of genes, totalling the complete set of eight genes, in *Saccharomyces cerevisiae*. Transformation of *S. cerevisiae* with the seven genes *easA* to *easG* genes resulted in the production of festuclavine, while transformation with the eight genes led to in the production of cycloclavine; these results suggested that *easH* is required for the last step of the cycloclavine biosynthetic pathway (Figure 4). This hypothesis was confirmed by testing the ratio of cycloclavine to festuclavine produced in the different yeast transformants. A good correlation between the copy number of *easH* and the production of cycloclavine versus festuclavine, was observed [7]. In vitro studies using combinations of *E. coli* expressed proteins: *EasA*, (the yellow enzyme), *EasG*, (the festuclavine synthase) and *EasH* (the cycloclavine synthase), proved that the three enzymes are required for the transformation of chanoclavine I aldehyde to cycloclavine; but the combination of the first two enzymes, EasA and EasG, resulted only in the formation of festuclavine. Studies on the enzymatic activity of the *easH*-encoded cycloclavine synthase showed that neither festuclavine nor agroclavine are real free intermediates in the biosynthesis of cycloclavine but rather they are side products of the cycloclavine pathway. Furthermore, when either pure festuclavine, agroclavine or chanoclavine-I-aldehyde were incubated with the cycloclavine synthase no cyclization reactions were observed, confirming that neither festuclavine nor agroclavine are real intermediates in the biosynthesis of cycloclavine. A putative intermediate, detectable by mass spectrometry(MS), (MS), which contains a double-bond in the fourth cycloclavine ring (Figure 4), has been proposed as the inmediate precursor of cycloclavine [7,8]. The cycloclavine synthase is a Fe^2+^ and α-ketoglutarate-dependent enzyme, a member of the phytanoyl-CoA hydroxylase family. Indeed, the Fe^2+^ cofactor and the α-ketoglutarate substrate were shown to be required for the in vitro conversion of chanoclavine I aldehyde to cycloclavine when the cycloclavine synthase was combined with the yellow enzyme. In absence of Fe^2+^ and α-ketoglutarate, the product of the combined enzymatic reaction was festuclavine but not cycloclavine, indicating that Fe^2+^ and α-ketoglutarate are required for the final conversion to cycloclavine. An interesting outcome of the research on the cycloclavine biosynthesis is the fact that *S. cerevisiae* transformant strains were developed that are able to produce relatively large amounts of cycloclavine. Previous studies [28] have shown that when the early genes of the pathway, involved in the formation of chanoclavine I, were expressed in *S. cerevisiae*, there was a limited conversion of *N*-methyl-4-dimethylallyl-l-tryptophan to chanoclavine I. When duplicated copies of the genes involved in this conversion were expressed in *S. cerevisiae* and in addition, all the late genes involved in the conversion of chanoclavine I to cycloclavine, were amplified in several copies, strains of *S. cerevisiae* able to produce up to 529 mg/L cycloclavine were obtained, a development that has industrial relevance. 

**Figure 5:**
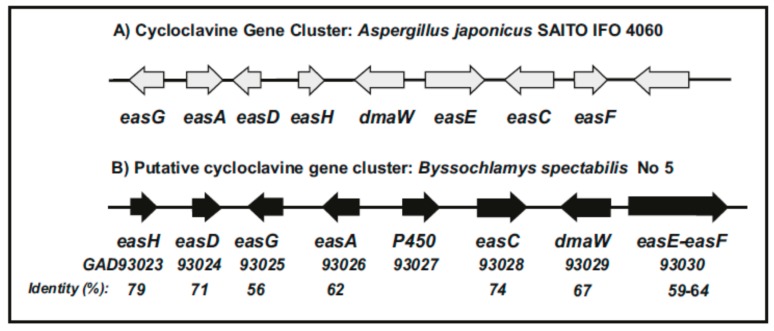
Cluster of homologous genes for clavine biosynthesis in *A. japonicus* and *B. spectabilis*. (**A**) Cluster of genes for cycloclavine biosynthesis in *A. japonicus*; (**B**) Putative gene cluster for cycloclavine biosynthesis in *B. spectabilis*. The name of the genes is indicated below the arrows. The accession number of the *B. spectabilis* genes, as well as the percentage of amino acids identity between the *B. spectabilis* and the *A. japonicus* homologous proteins is indicated.

In a search of putative homologs of *easH* in the currently available fungal gene banks only a highly similar gene (79% identity to EasH) was found in *B. spectabilis* (anamorph *Paecelomyces variotii*), suggesting that this fungus may produce cycloclavine. A detailed analysis of the region containing the cycloclavine synthase gene *easH* of *B. spectabilis* revealed that there is a complete gene cluster for the biosynthesis of cycloclavine (GAD93023 to GAD93030) [44], including genes for all steps of the biosynthetic pathway (Figure 5), although the order of the genes is different from that of *A. japonicus*. Interestingly, the genes encoding the oxidoreductase (*easE*) and the gene for the *N*-methyl-4-dimethylallyl-l-tryptophan transferase (*easF*) appear to be fused in *B. spectabilis*, as found also in *P. steckii* and both fused genes have a 56% identity (see below), although the possibility of erroneous delimitation of the ORF’s in the data bases can not be excluded. Analysis of the encoded protein’s and their functionality is required to clarify this point. In addition, in the *B. spectabilis* cluster there is a P450 monooxygenase gene, which is not present in the *A. japonicus* cluster. However, it is unclear at this time if detectable amounts of cycloclavine are produced by *B. spectabilis*.

## 5. A Chanoclavine I Gene Cluster Exist in Members of the Arthrodermataceae Family

Members of the Arthrodermataceae fungi are important because they infect keratinized skin tissues and produce dermatomycosis in human and animals. The most relevant dermatophytic fungi include species of the genera *Arthroderma* and *Trichophyton*. Using the genome sequences developed by the Arthroderma Genome Sequencing Consortium [45], a five-gene cluster in *Arthroderma benhamiae*, *Arthroderma otae* and *Trichophyton verrucosum* has been identified [9] and more recently in some other *Trichophyton* species [41]. Bioinformatic analysis of the nucleotide sequences identified genes that were similar to the clavine orthologous genes in *N. fumigata* and to the homologous genes involved in the biosynthesis of ergot alkaloids in *C. purpurea*. The five genes correspond to those known to be involved in the conversion of tryptophan and dimethylallyl-pyrophosphate into chanoclavine I aldehyde, i.e., the core steps common to all clavine and ergot alkaloid producing fungi. The conservation of the encoded proteins was quite high between *Arthroderma* and *Trichophyton*, with amino acid identities between 70% and 92% [9], suggesting that the clusters in these two genera are closely evolutionarily related; they are distant from the gene clusters present in the Aspergillaceae or Clavicipitaceae fungi. No late genes for the conversion of chanoclavine I aldehyde to other putative alkaloids have been found in the vicinity of the five genes cluster; however, Wallwey et al. [9] did not exclude the possibility that other genes are located elsewhere in the genome of these dermatophytic fungi. Indeed, we have found in the genome of *Arthroderma benhamie* genes encoding enzymes similar to the yellow enzyme and to the festuclavine synthase (XP_003015710, XP_003014997) with 40% and 41% identity to the homologous enzymes of *N. fumigata*. The presence of these genes outside of the five genes cluster is relevant on the light of recent advances on the location of biosynthetic genes divided into subclusters in *P. roqueforti* and other fungi [10,11]. 

The chanoclavine dehydrogenase ChaDH was expressed in *E. coli*, purified by affinity chromatography and tested in vitro [9]. Using NAD^+^ as cofactor ChaDH was able to convert the substrate chanoclavine I into chanoclavine I aldehyde, confirming its in vitro functionality. The requirement of NAD^+^ as cofactor was specific since it could not be replaced by NADP^+^, FMN or FAD as proton acceptor. Interestingly, *A. benhamiae* shows no significant production of chanoclavine I aldehyde when grown in several culture media, so the final product of the pathway is still unclear; chanoclavine I aldehyde might be converted into other uncharacterized final products. Wallwey and coworkers [9] tested the expression of the five genes of the *A. benhamiae* cluster and found that expression of these genes was extremely low; it is well known that many genes for secondary metabolites are not expressed or are expressed very poorly in different nutritional or environmental conditions [46,47]. 

## 6. Role of Non-Clustered Non-Ribosomal Peptide Synthetases on Fumigaclavine C Biosynthesis

An intriguing observation is the finding that in *N. fumigata* mutants blocked in two different non-ribosomal peptide synthetases (NRPSs) that are not linked to the fumigaclavine C gene cluster are required for the biosynthesis of this toxin. The two NRPSs PesL and Pes1 have been studied by O’Hanlon et al. [48]. The first, PesL, is a monodomain NRPSs that has been reported to be involved in the biosynthesis of fumiquinazoline [49] and Pes1 is a multimodular non-lineal NRPSs of unknown function. Disruption of any of these two NRPSs blocks the conversion of fumigaclavine A to fumigaclavine C, which is catalyzed in *N. fumigata* by the reverse prenyltransferase FgaPT1. The conversion of fumigaclavine A into fumigaclavine C, has been experimentally confirmed in vitro in *N. fumigata* using purified prenyltransferase FagPT1 [23] and therefore it is difficult to understand how NRPSs, located outside of the fumigaclavine cluster are required for fumigaclavine C biosynthesis. The early and middle steps in the fumigaclavine biosynthesis are not apparently affected in these mutants, as shown by the fact that they still synthesized fumigaclavine A. The mutants blocked in PesL or Pes1 show increased formation of other alkaloid toxins such as fumitremorgin C and verruculogen. The exact mode of action of these two NRPSs is obscure. They apparently act by helping the prenyltransferase FgaPT1 to perform the prenylation step in vivo. These NRPSs might be involved in the activation and/or transfer of intermediates; alternatively, these NRPSs may act as chaperons for the clavine biosynthetic enzymes. In fact, there are some other NRPSs in filamentous fungi that affect the biosynthesis of compounds, for which they play no clear role in their biosynthetic pathways, apparently by a cross-regulation mechanism [50,51]. In *P. roqueforti* there are homologues to Pes1 and PesL with moderate identity to these NRPSs (35–46% identity) but even if these NRPSs are present in *P. roqueforti*, this strain is unable to produce isofumigaclavine C because of the absence of a reverse prenyltransferase similar to FgaPT1.

## 7. The *Penicillium* and Other Aspergillaceae and Arthrodermataceae Fungi Lack the Conversion of Agroclavine to Lysergic Acid

The *Claviceps* species and the endophytic *Epichloë* species synthesize lysergic acid, a molecule that contains an unsaturated double bond between carbons C-9 and C-10 and is oxidized at carbon C-17. On the other hand, the festuclavine-type clavine alkaloids contains a saturated ergoline B ring and the methyl group at carbon C-17 is fully reduced (Figure 1). The conversion of agroclavine to lysergic acid involves: (i) the cumulative transfer of six electrons during oxidation of the methyl group at C-17; and (ii) the isomerization of a double bond between carbons C-8 and C-9, that occurs in agroclavine and elymoclavine, to carbons C-9 and C-10 that is present in lysergic acid. Branching of the pathways for the biosynthesis of clavine and ergot alkaloids takes place at the chanoclavine I aldehyde intermediate [3,14]. Chanoclavine I aldehyde is converted in festuclavine in the clavine alkaloids but in the Clavicipitaceae fungi is transformed into lysergic acid through the intermediate agroclavine. Early studies on the biosynthesis of the ergot alkaloids in *C. purpurea* showed that agroclavine may be converted through the intermediate elymoclavine into paspalic acid or lysergic acid. A P450 monoxygenase encoding gene, named *cloA* (for clavine oxidase), was found to be involved in the conversion of agroclavine to lysergic acid. This P450 monoxygenase is known to catalyze the oxidation of the methyl group at C-17 of elymoclavine to lysergic acid [52]. The ability to synthesize these two compounds was recovered by transformation of the disrupted mutant with the native *cloA* allele. The CloA enzyme is present in the ergot alkaloid producing Clavicipitaceae fungi but is absent in clavin alkaloids producing fungi. Indeed, our studies reveal that *P. roqueforti* lack a gene encoding an enzyme orthologous to CloA. Disruption of *cloA* led to the accumulation of elymoclavine and agroclavine and lack of formation of lysergic acid or paspalic acid. These results were later confirmed by heterologous expression in *N. fumigata* of a combination of the two *C. purpurea* genes, *cloA* and *easA* that is required for agroclavine formation from chanoclavine I aldehyde [25]. The combination of the two genes introduced allowed the synthesis first of agroclavine and then of lysergic acid. This engeneered *N. fumigata* strain, carrying the *cloA* gene, does not secrete the intermediate elymoclavine, suggesting that the CloA enzyme is able to carry a multistep reaction without releasing this intermediate.

## 8. Evolutionary Relationship between Clavine and Ergot Alkaloids: A Recruiting Play of Prenyltransferases, Monooxygenases and Dioxygenases

Accumulated evidence in the last few years, based on the availability of a large number of genome sequences, shows that differences in the last steps of the biosynthesis of clavine and ergot alkaloid producer fungi is largely due to a combination of activities of several enzymes, usually encoded in the respective gene clusters. These include prenyltransferases of the reverse type, dioxygenases of the phytanoyl CoA-hydroxylase class and P450 monooxygenases.

### 8.1. Gene Gains, Gene Losses and Gene Rearrangement in the Clavine Alkaloid Producers

An interesting case of gene losses or gene gains is the difference in the last step of the fumigaclavine C biosynthetic pathway, catalized by the reverse prenyltransferase *fgaPT1*. This gene is absent in *P. roqueforti* and all other *Penicillium* studied but is present in *N. fumigata*. Interestingly, several *N. fumigata* wild type strains isolated from natural habitats have partially deleted the *fgaPT1* gene and in some others, which not show *fgaPT1* deletion, the gene is not expressed [11]. This finding suggests that, indeed, *fgaPT1* is lost or rearranged with certain frequency in nature and this may explain the lack of this gene in *Penicillium* species [10].

Another significant difference is the presence of a gene for the oxidation of festuclavine to form fumigaclavine B (oxidation at C-9) that occurs in *N. fumigata* and *P. commune* but is absent in *P. roqueforti*, although other P450 monooxygenases appear to be able to convert festuclavine to isofumigaclavines B and A, since *P. roqueforti* is known to produce these compounds. Differences in the organization of the gene clusters are also evident between *N. fumigata* and *Penicillium* species as indicated in Figure 2. In particular, is relevant the presence in *P. roqueforti* of two copies of the festuclavine synthase gene, *ifgF1* and *ifgF2*, since neither *N. fumigata* nor *P. commune* have been described to contain this gene duplication. It is likely that the absence of a festuclavine hydroxylase in *P. roqueforti* has resulted from a rearrangement of the isofumigaclavine gene cluster that is present in *P. commune* and *N. fumigata* that gives rise to the divided clusters in *P. roqueforti*.

The presence of duplicated fumigaclavine synthase genes in *P. roqueforti* is interesting. Comparison of the clavine clusters in *P. roqueforti* and *N. fumigata* and other *Penicillium* species, indicates that this duplication occurred recently in *P. roqueforti*, since it is not present in the clusters of other *Penicillium* species. Perhaps one of the duplicated copies of the fumigaclavine synthase is responsible for the formation of a festuclavine steroisomer with the 8S/9R configuration that is later converted to isofumigaclavine A, whereas the other copy may be required for the formation of festuclavine, since the normal configuration of festuclavine is 8S/9S. This implies that an isomer of festuclavine with the 8S/9R configuration, isofestuclavine, may exist in *P. roqueforti*. It is known that in addition to festuclavine *P. commune* produce small amounts of pyroclavine, the isomer 8R/9S [41]. 

### 8.2. Plasticity of the Clusters: Clavine Gene Clusters in Penicillium Species

There are more than three hundred *Penicillium* species known [53] and an important point is how many of these species have the clavine gene cluster. We have compared the putative gene clusters in *P. camemberti*, *P. expansum*, *P. steckii* and *P. griseofulvum* and we found genes related to the clavine gene cluster in these four species. Most of these clusters appear to be non-functional, because they lack some of the essential genes that occur in *P. roqueforti* and *P. commune*. 

#### 8.2.1. Clavine Gene Cluster in *Penicillium camemberti*

In *P. camemberti* there is a single cluster (CRL19773-CRL19778) containing in the following order genes homologous to *phaA*, *ifgD*, *ifgB*, *ifgC*, *ifgA* and *ifgE* [32]. The phytanoyl-CoA hydroxylase of *P. camemberti* has 55% identity to *easH* of the *A. japonicus* and 36 % to the phytanoyl CoA hydroxylase of *P. roqueforti* clavine gene cluster. In *P. camemberti* we cannot find a gene similar to the festuclavine hydroxylase, suggesting that this cluster is defective in clavine production; indeed *P. camemberti* has not been described as a clavine producer [54]. *P. camemberti* contains genes similar to *pesL* and *pes1*, (CRL19476 and CRL18780) with 57% and 45% identity to the homologous genes of *N. fumigata*, respectively, that are located far from the clavine cluster, as occurs in *N. fumigata*. There is a yellow enzyme encoding gene (50% identity to IfgG) but is not located in the clavine cluster, either. Adjacent to the clavine gene cluster is a gene (CRL19781) encoding a Zn(II)_2_-Cys_6_ fungal type DNA binding protein, a regulatory protein that has been found associated with control of gene expression in other fungal gene clusters. 

#### 8.2.2. Clavine Gene Cluster in *Penicillium expansum*

In *P. expansum* there are two clusters: (1) XP_016600807 to XP_016600817; and (2) XP_016594733 to XP_016594736 [55]. The first cluster carries in the following order genes homologous to *ifgC*, *ifgD*, *ifgA* and genes for a P450 monooxygenase, a phytanoyl-CoA hydroxylase and an acetyltransferase. Other genes unrelated to clavine biosynthesis are inserted in cluster 1. In the second cluster, we found genes homologous to *ifgE*, *ifgG*, *ifgF* and *ifgB*. The gene encoding an acetyltransferase in cluster 1, while similar to *ifgI* (31% identity) encodes a protein similar to feruloyltransferases, larger than the common acetyltransferases found in clavine clusters. The phytanoyl-CoA hydroxylase has low identity (26% identity) to *easH* of *A. japonicus* and lower to the phytanoyl-CoA hydroxylase of *P. roqueforti*. 

#### 8.2.3. Clavine Gene Cluster in *Penicillium steckii*

In *P. steckii* clavine biosynthesis genes are located in a single cluster (genes OQE13742 to OQE13753). It contains in the following order homologous to: *ifgA*, *ifgE*, *ifgD*, *ifgB* and *ifgF*, but other genes there are inserted between them [53]. Surprisingly, *P. steckii* OQE13752 (encoding a 920 amino acid protein) is a fused gene. The protein encoded shows identity with the FAD-dependent oxidoreductase IfgC in the N-terminal moiety (69%) and with the *N*-methyltransferase of DMAT, IfgB in the C-terminal region (80%). This hybrid protein is end-to-end similar to the hybrid oxidoreductase/*N*-methyltransferase of *B. spectabilis*. In *P. steckii* genome there are genes for a phytanoyl-CoA hydroxylase (OQE19090, 76% identity with *P. roqueforti pahA*), a single gene for an acetyltransferase (OQE21692, 50% identity to *ifgI*), genes for P450 (less than 30% identity with *N. fumigata* homologous protein) and two genes similar to IfgG (49% and 60% identity) of *P. roqueforti* but all of them are scattered and far from the clavine cluster.

#### 8.2.4. Clavine Gene Cluster in *Penicillium griseofulvum*

In *P. griseofulvum* four genes, homologous to *ifgI*, *ifgC*, *ifgD* and *ifgA* (KXG48657 to KXG48664) and a gene for a P450 monooxygenase, with low identity to *fga*Ox2 of *N. fumigata*, are linked together in a single cluster [56] and five genes, homologous to the remaining genes of the clavine pathway are disperse in the genome with identities ranging from 35 to 54% in relation to *P. roqueforti* genes. This species was reported many years ago to produce chanoclavin I, although its biosynthesis has not been studied [57] indicating that the clavine cluster in this fungus is functional at least up to the chanoclavine I step. The lack of late intermediates and final products in this fungus correlates well with the absence of a chanoclavine I dehydrogenase gene in the gene cluster.

### 8.3. Role and Evolution of Phytanoyl-CoA Hydroxylase Enzymes in Clavine and Ergot Alkaloid Biosynthesis

A gene encoding a protein belonging to the family of phytanoyl-CoA hydroxylase (CDM30152, named *pahA*) is located in cluster B of *P. roqueforti* [10]. Notably, there are genes encoding phytanoyl-CoA hydroxylases in several clavine and ergot alkaloid gene clusters. In *N. fumigata*, this gene is located outside of the fumigaclavine gene cluster and the identity of the encoded protein with the PahA protein of *P. roqueforti* is low. 

There is a gene similar to phytanoyl-CoA hydroxylases in the ergot cluster of *C. purpurea*; it encodes a protein involved in the modification of the first amino acid of the tripeptide chain of ergopeptines [37]. However, in *P. roqueforti* there is no formation of lysergic acid or lysergic acid derived ergotamines and the possible hydroxylation step in *P. roqueforti* is unclear. Another example of members of the phytanoyl-CoA hydroxylase family that is related to clavine alkaloids biosynthesis is the cycloclavine synthase that has been described above. *P. roqueforti* does not forms cycloclavine and lacks an authentic cycloclavine synthase. The relationship between the phytanoyl-CoA hydroxylase of *P. roqueforti* and EasH (cycloclavine synthase) of *A. japonicus* is distant (34% identity), suggesting that these enzymes have diverged to adapt to different biosynthetic hydroxylation functions in *P. roqueforti* and *A. japonicus*.

An additional pseudogene with an open reading frame of small size (121 amino acids, CDM30154), different from the *pahA* gene (CDM230152) has been found in the clavine cluster B of *P. roqueforti*. Surprisingly, the amino acid sequence encoded by this small gene-fragment is 56% identical to an internal sequence of the EasH protein of *A. japonicus*. This high identity indicates that the rearrangement of a progenitor gene has occured in *P. roqueforti* and that the small open reading frame is a remnant of an ancestral *easH* gene. 

In summary, from the evolution point of view the presence of genes for related phytanoyl-CoA hydroxylase in several clavine and ergot alkaloid gene clusters suggests that a progenitor enzyme of this class has been adapted to a different function in the clavine and ergot alkaloids biosynthetic pathways. 

### 8.4. Vertical Gene Diversification versus Horizontal Gene Transfer

Although the relationship between the biosynthetic pathways of ergot alkaloids in the Clavicipitaceae fungi and the clavine alkaloids in Aspergillaceae fungi is clear, the low identity of the enzymes in both fungal groups at the amino acid level suggests that there is a significant degree of divergence in the evolution of these gene clusters. Particularly this is more evident in the late genes of the pathways, which are different in the ergot alkaloid producers and the clavine producers. This is also relevant in the Arthrodermataceae fungi which appear to lack functional late genes involved in the conversion of chanoclavine I aldehyde to the end product of the clavine pathway. One of the more important differences is the presence in Clavicipitaceae fungi of NRPSs involved in the formation of the peptide chains of the lysergic acid derived ergopeptines. The absence of genes for the peptide synthesizing enzymes, LPS1/LPS2, in the gene cluster of *Penicillium* species agrees with the lack the CloA enzyme require for the formation of lysergic acid, as described above. The clavine clusters of the Arthrodermataceae fungi are related to the clavine alkaloid clusterd in *Penicillium* and *Neosartorya* and more distant to the the ergot alkaloid clusters in the Clavicipitaceae fungi. The accumulated scientific evidence suggests that both groups of alkaloid gene clusters derive from a very ancient common progenitor cluster that has diverge over millions of years. This explains the low identity between genes which are common to both types of alkaloid producing fungi, although a horizontal gene transfer cannot be excluded at an early stage that has later diverged [41]. A likely example of horizontal gene transfer is the presence of a complete cycloclavine gene cluster in *B. spectabilis*, that may have been transferred from *A. japonicus* and rearranged (Figure 5). *B. spectabilis* belongs to the Thermoascaceae family, phylogenetically distant from the Aspergillaceae family and therefore a possible horizontal gene transfer may explain the presence of this cluster in these distantly related genera. Indeed, Nielsen et al. [53] in a study of the genome of several *Penicillium* species proposed that the gene divergence and horizontal gene transfer are more general, affecting also polyketides and non-ribosomal peptides gene clusters. Similar observations were also made by Firkpatrick [58] in different fungi. 

## Figures and Tables

**Table 1:** Function of the *ifg* genes, located in the isofumigaclavin gene cluster of *Penicillium roqueforti*. Comparison of the encoded proteins with orthologous/homologous proteins in other clavine clusters.

Gene Name in *P. roqueforti* ^1^	Function	*N. fumigata* (%) ^2^	*A. japonicus* (%)	*P. camemberti* (%)	*P. expansum* (%)	*P. steckii* (%)	*P. griseofulvum* (%)
*ifgA*, CDM36678	DMAT	*fga*PT2, *dmaW* (64%)	*dmaW* (66%)	CRL19777 (84%)	XP_16600812 (60%)	OQE13746 (83%)	KXG48664 (60%)
*ifgB*, CDM36676	Methyl Transferase	*fga*MT, *easF* (63%)	*easF* (66%)	CRL19775 (85%)	XP_16604736 (53%)	OQE13752 (80%)	
*ifgC*, CDM36677	FAD oxidase	*fga*Ox1, *easE* (49%)	*easE* (48%)	CRL19776 (77%)	XP_16600807 (47%)	OQE13752 (69%)	KXG48659 (44%)
*ifgD*, CDM36675	Catalase	*fga*Cat, *easC* (62%)	*easC* (61%)	CRL19774 (90%)	XP_16600810 (63%)	OQE13751 (84%)	KXG48662 (60%)
*ifgE*, CDM36673	Short Chain DH	*fga*DH, *easD* (72%)	*easD* (67%)	CRL19778 (66%)	XP_16604733 (67%)	OQE13747 (68%)	
*ifgG*, CDM30151	Yellow Enzyme	*fga*Ox3, *eas*A (67%)	*easA* (64%)	CRL20441 (50%)			
*ifgF1*, CDM36674	Festuclavine Synthase I	*fga*SF, *eas*G (60%)	*easG* (54%)		XP_16604735 (49%)	OQE13753 (58%)	
*ifgF2*, CDM30155	Festuclavine Synthase II ^3^						
*ifgI*, CDM30153	Acetyltransferase	*fga*AT, *eas*N (32%)					KXG 48657 (30%)
	Festuclavine Hydroxylase (P450) ^4^	*fga*Ox2, *eas*M					
*pahA*, CDM30152	Phytanoyl-CoA Hydroxylase		*easH* (34%)	CRL19773 (36%)			

^1^ The *ifg* genes (A to I) are named according to the sequential enzymatic steps in the pathway; ^2^ Percentage of amino acids identity in the encoded protein in relation to the orthologous/homologous proteins of *P. roqueforti*; ^3^ Only *P. roqueforti* has two festuclavine synthases; ^4^ The festuclavine hydroxylase gene is present only in the fumigaclavine gene cluster of *N. fumigata*.
